# High-Grade Mucinous Adenocarcinoma of the Appendix With Signet Features and Omental Metastasis

**DOI:** 10.7759/cureus.31525

**Published:** 2022-11-15

**Authors:** Fady Banno, Brandon Wiggins, Kyle T Knight, Kanksha Peddi, Ali Alrammahi, Nathan Landesman

**Affiliations:** 1 Internal Medicine, Ascension Genesys Hospital, Grand Blanc, USA; 2 Pathology, Ascension St. John Providence, Detroit, USA; 3 Gastroenterology, Ascension Genesys Hospital, Grand Blanc, USA

**Keywords:** gastroenetrology, internal medicine, gi cancers, signet ring cell adenocarcinoma, mucinous adenocarcinoma of the appendix

## Abstract

Primary adenocarcinoma of the appendix is an uncommon malignancy of the gastrointestinal tract. The most common symptom is right lower abdominal pain, which could be indistinguishable from acute appendicitis. However, the clinical presentation is usually non-specific. In this present report, we describe a rare case of primary signet ring cell carcinoma of the appendix with omental metastases in an 81-year-old male who underwent laparoscopic appendectomy. He then received palliative systemic chemotherapy using a combination of Capecitabine and Bevacizumab.

## Introduction

Primary adenocarcinoma of the appendix is an uncommon malignancy of the gastrointestinal (GI) tract. It was first described in 1882 and constitutes around 0.12 to 2.6 cases per one million people per year [1]. In addition, signet-ring cell carcinoma of the appendix is extremely rare, it constitutes approximately 4% of all primary appendiceal neoplasms [2].

The most common symptom is right lower abdominal pain, which could be indistinguishable from acute appendicitis. However, the clinical presentation is usually non-specific [3]. The diagnosis of signet cell adenocarcinoma is confirmed after surgical excision of the inflamed appendix and pathological analysis. The current treatment options for the metastatic disease include systemic chemotherapy, cytoreductive surgery with peritonectomy, and/or hyperthermic intraperitoneal chemotherapy (HIPEC).

There are few reported cases of primary signet cell appendiceal carcinoma. In this present report, we describe a rare case of primary signet ring cell carcinoma of the appendix with omental metastases in an 81-year-old male who underwent laparoscopic appendectomy. He then received palliative systemic chemotherapy using a combination of Capecitabine and Bevacizumab.

## Case presentation

**Figure 1:**
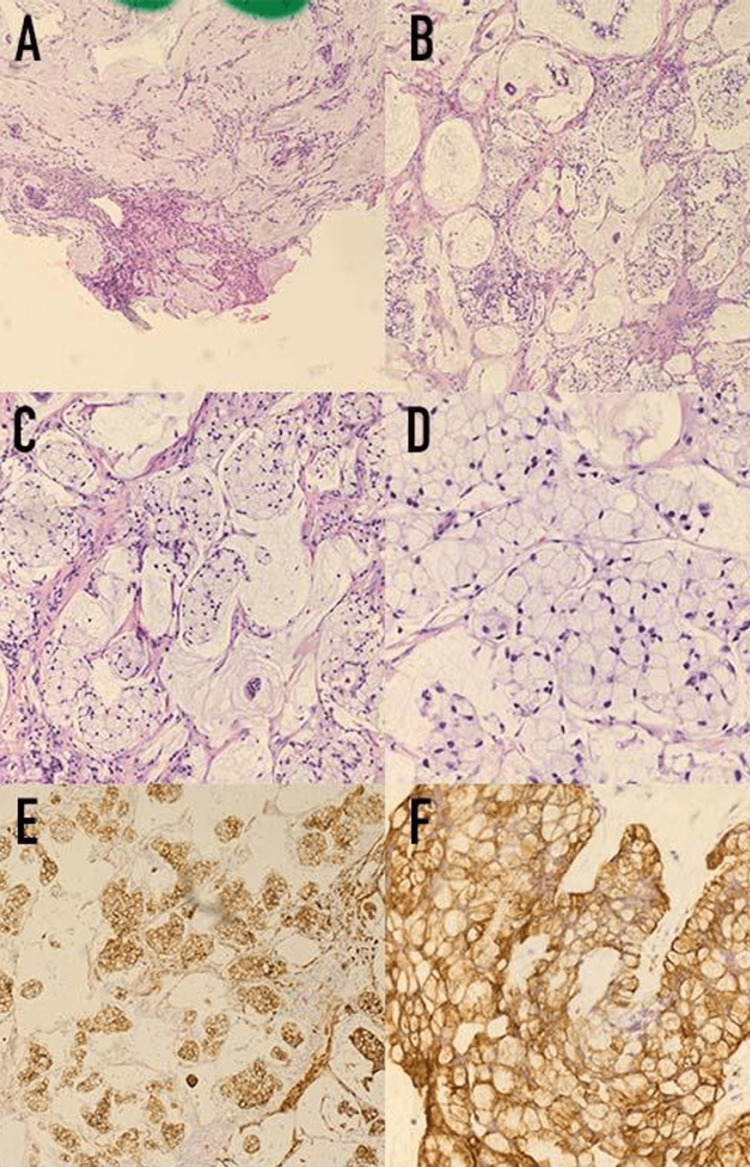
Histologic hematoxylin and eosin (H&E) examination showing a signet cell carcinoma of the appendix infiltrating the peritoneum and diaphragm. (A) Right lower quadrant peritoneum biopsy, (H&E stain 100x): High-grade mucinous carcinoma peritonei with signet ring cells. The pool of mucin with a cluster of epithelium that shows signs of metastatic carcinoma (enlarged nuclei, atypia, pleomorphic changes). (B-D) Right diaphragm peritoneum biopsy. High grade mucinous carcinoma peritonei with signet ring cells. (B) at 100x, (C) at 200x, and (D) at 400x. (E, F) Immunohistochemical cytokeratin stain (AE1/AE3) shows strong positivity of high-grade carcinoma. (E) at 100x, and (F) at 400x.

We present a case of an 81-year-old male with a past medical history of hypertension, hyperlipidemia, degenerative joint disease, depression, pulmonary fibrosis, benign prostatic hypertrophy, and prior negative esophagogastroduodenoscopy (EGD) and colonoscopy in 2017 who presented to an urgent care in January 2021 with abdominal pain. Lab work was unremarkable, and the patient was sent home with instructions to visit the emergency department (ED) if he experienced fever, increased abdominal pain, or blood in the stool. The patient presented to the emergency department with increased abdominal pain in April 2021, and Computed tomography (CT) scan of the abdomen and pelvis with intravenous contrast showed a cystic appendiceal lesion with right peritoneal nodularity suspicious for peritoneal carcinomatosis. Therefore, the patient underwent an appendectomy the following day revealing poorly differentiated, high-grade appendiceal adenocarcinoma with signet ring features, pT4a disease (Figures 1A-1F).

A follow-up CT scan of the chest, abdomen, and pelvis one month later showed an enlarged carinal lymph node measuring 2.4 x 1.6 cm with diffuse omental infiltration suggestive of peritoneal carcinomatosis. In addition, tumor markers were checked, and carcinoembryonic antigen (CEA) was elevated to 42.6 µg/L. The patient had a diagnostic laparoscopy in June 2021 and was found to have diffuse peritoneal carcinomatosis. Multiple biopsies were obtained and came back positive with high-grade mucinous adenocarcinoma with signet ring features. The patient agreed to pursue systemic chemotherapy. He was initiated on Capecitabine (Xeloda) 2,000 mg tab PO, twice daily for 14 days and off for seven days. Bevacizumab (Avastin) 7.5 mg IV infusion every three weeks.

In September 2021, he presented to the ED again complaining of four out of 10, dull, diffuse abdominal pain. Lab work was significant for elevated lactic acid at 3.7 mmol/L with a negative Clostridium difficile test. CT abdomen/pelvis showed unchanged nodular omental metastasis, and his abdominal pain was attributed to Xeloda as well as metastatic cancer. The patient deteriorated during his admission with rising lactic acid levels in addition to worsening respiratory function due to underlying pulmonary fibrosis. The patient was do not resuscitate/do not intubate (DNR/DNI) and passed away after a lengthy admission.

## Discussion

Appendiceal malignancies are very rare, and incidence has been rising since the turn of the 21st century - likely related to improved access to healthcare, imaging, surveillance, and awareness of the disease process [4,5]. Appendiceal neoplasms make up only 0.5% to 1% of all intestinal cancers, with signet cell pathology in even fewer cases [6]. Of the 1% of appendiceal intestinal malignancies, 66% are carcinoid in nature, and adenocarcinoma makes up the remaining portion [7].

Appendiceal malignancies and specifically signet ring adenocarcinoma typically secrete mucin, leading to metastasis throughout the peritoneum and eventual obstruction [8]. This has been termed pseudomyxoma peritonei (“Jelly Belly”), which histologically spans from high-grade, poorly differentiated signet cell tumors to low-grade, well-differentiated neoplasms. They are classified pathologically as either mucinous/intestinal type or non-mucinous/colonic type depending on the amount of mucin seen in tumor samples, with over 50% of mucin samples being designated as mucinous. Mucinous adenocarcinoma of the appendix is usually characterized by large quantities of invasive tumor cells with associated desmoplastic stromal reactivity [9].

Immunochemistry is especially helpful when classifying colorectal tumors, including appendiceal malignancies. Appendiceal malignancies can sometimes be mistaken for ovarian cancer and are differentiated by their genetic markers via CK20, CK7, and CDX2 [10].

Patients with appendiceal neoplasms may have vague lower abdominal pain but are usually asymptomatic with an incidental diagnosis on imaging or appendectomy [11]. Other possible presentations include GI bleeding due to local invasion into the lumen of the GI tract and/or obstruction due to the mucinous nature of the tumors [12]. Labs can possibly be significant for anemia, leukocytosis, and/or CEA elevation [13].

Diagnosis can be assisted with CT, magnetic resonance imaging, barium enema, ultrasound, or endoscopy. Diagnosis is confirmed upon biopsy of the tumor, metastatic lesion, or appendectomy (if the lesion is confined to the appendix) [14]. Due to the high risk of peritoneal spread, a biopsy of the primary tumor should be avoided whenever possible, and appendectomy or peritoneal metastasis sampling is preferred [15].

Treatment is dictated by the extent of the disease, including metastasis, as well as tumor integrity. Localized malignancy should be treated with appendectomy, especially for diagnostic purposes, as biopsy may cause seeding. If there is a concern for spread/rupture, extended bowel resection, HIPEC, and/or cytoreductive surgery may be required [16]. For patients with nodal metastasis, adjuvant FOLFOX (leucovorin calcium, fluorouracil, and oxaliplatin) or CAPOX (capecitabine and oxaliplatin) regimens are recommended [17]. Radiation therapy is controversial and has not been studied enough for the recommendation. Further gene-targeted therapies are presently being researched, and recently epidermal growth factor receptor (EGFR) positive cancers have been treated with bevacizumab, a vascular endothelial growth factor inhibitor [18].

The prognosis for mucinous adenocarcinomas of the appendix is poor, with higher morbidity/mortality in those with signet cell pathology. Five-year survival for mucinous adenocarcinomas is 53.6% with five-year mortality increasing to 90% for stage IV poorly differentiated adenocarcinoma, typically the signet cell type [19].

## Conclusions

Appendiceal malignancies are very rare. Signet ring adenocarcinoma of the appendix typically secretes mucin leading to metastasis throughout the peritoneum and eventual obstruction. Treatment is based on stage and histology. Surgical resection and peritoneal debulking are possible. High-grade tumors require further prospective trials to evaluate treatment, but treatment options include surgical management, HIPEC, and systemic chemotherapy.
